# Shedding Light on the Extended Mind: HoloLens, Holograms, and Internet-Extended Knowledge

**DOI:** 10.3389/fpsyg.2021.675184

**Published:** 2021-10-21

**Authors:** Paul R. Smart

**Affiliations:** Electronics and Computer Science, University of Southampton, Southampton, United Kingdom

**Keywords:** active externalism, extended mind, extended knowledge, Internet, mixed reality, HoloLens

## Abstract

The application of extended mind theory to the Internet and Web yields the possibility of *Internet-extended knowledge*—a form of extended knowledge that arises as a result of an individual's interactions with the online environment. The present paper seeks to advance our understanding of Internet-extended knowledge by describing the functionality of a real-world application, called the HoloArt app. In part, the goal of the paper is illustrative: it is intended to show how recent advances in mixed reality, cloud-computing, and machine intelligence might be combined so as to yield a putative case of Internet-extended knowledge. Beyond this, however, the paper is intended to support the philosophical effort to understand the notions of extended knowledge and the extended mind. In particular, the HoloArt app raises questions about the universality of some of the criteria that have been used to evaluate putative cases of cognitive extension. The upshot is a better appreciation of the way in which claims about extended knowledge and the extended mind might be affected by a consideration of technologically-advanced resources.

## 1. Introduction

According to proponents of the extended mind, human mental states and processes are, on occasion, subject to a form of wide or extended realization, such that resources lying beyond the ancient metabolic boundaries of skin and skull are included as part of the physical fabric that realizes human mental states and processes. In short, the idea is that “the causally-active physical vehicles of content and of cognitive process [can] be spread across the biological organism and the world” (Clark, 2010b, p. 45).

The *locus classicus* for work on the extended mind is a paper by Clark and Chalmers (1998). As part of that paper, Clark and Chalmers describe a thought experiment involving two individuals, named Otto and Inga, who are on a trip to New York City. We are asked to imagine that Otto and Inga wish to visit The Museum of Modern Art (MOMA), which is located on 53rd Street. Inga is a neurologically unimpaired individual who relies on bio-memory to retrieve information about the museum's location. Otto, by contrast, is a memory-impaired individual who relies on a paper-based notebook to retrieve information about the museum's location. Despite the fact that Otto and Inga rely on different ways of retrieving the relevant information—Inga relies on a brain-based neural circuit, while Otto relies on a world-involving circuit—Clark and Chalmers (1998) suggest that both Otto and Inga ought to be credited with the standing or dispositional belief that MOMA is located on 53rd Street. As noted by Clark (2011), the Otto/Inga thought experiment (also known as the Otto case) was:

…meant to convince the reader that, under certain conditions, the coarse functional role of a bio-external encoding could be sufficiently similar to that of a persisting internal encoding as to mandate similar treatment, revealing the non-biological resource as part of the physical machinery underpinning some of an agent's genuine mental states. (Clark, 2011, p. 448)

The Otto case is founded on a relatively simple bio-external resource, namely, a paper-based notebook. In recent years, however, arguments for the extended mind have been applied to a number of technologically-advanced resources (Record and Miller, 2018; Smart, 2018; Pedersen and Bjerring, forthcoming). Of particular interest is the extent to which the informational and technological elements of the online environment (the Internet and Web) can support extended minds, thereby yielding the possibility of so-called Web- or Internet-extended minds (Smart, 2012, 2017). These debates have also started to converge with a parallel stream of research relating to the epistemological implications of the extended mind, a branch of epistemology known as extended epistemology (Pritchard, 2010, 2018; Carter et al., 2018a). The result is a growing preoccupation with the epistemic significance of the Internet and Web, as seen from the standpoint of claims about the extended mind. The practical implications of this sort of work should be clear. If we accept that online information can play the same sort of functional role as that served by the information in Otto's notebook, then it seems that the Internet-extended cognizer could be subject to a significant expansion in their body of dispositional beliefs (see Ludwig, 2015). Indeed, assuming that such dispositional beliefs are true, we may wonder whether this form of doxastic expansion entails a corresponding form of epistemic expansion, such that human individuals can come to enjoy various forms of restricted omniscience—a “complete, or close to complete knowledge about a particular, fairly specific subject matter” (see Bjerring and Pedersen, 2014, p. 25). The application of extended mind theory to the online environment thus yields the possibility of *Internet-extended knowledge*—a form of extended knowledge in which the Internet and Web are (as Clark suggests) “part of the physical machinery underpinning some of an agent's genuine mental states.”

The present paper describes a putative case of Internet-extended knowledge that combines the use of online processing with the forms of visualization and interaction enabled by a mixed reality device, namely, the Microsoft HoloLens (see section 2). The aim is to show how a range of contemporary digital technologies might be used to satisfy some of the criteria that have emerged in respect of the notion of extended knowledge. These include criteria relating to the reliability of belief-forming processes (see section 3), as well as criteria relating to issues of accessibility and trust (see section 4). As an added bonus, the paper shows how a consideration of advanced technologies introduces us to issues that were not part of the original Otto case (see section 5). This is important, for it raises questions about the universality of criteria that have been used to evaluate putative cases of cognitive/epistemic extension: Are these criteria universally applicable to all forms of cognitive/epistemic extension, or are they more a product of the specific features of the original Otto case (e.g., the properties of Otto's notebook)? The present paper marks the beginnings of an attempt to explore this issue by examining cognitive/epistemic extension in a technologically-advanced setting.

The paper has two broad, overarching aims. The first is to provide a practical demonstration of the effort to support Internet-extended knowledge. This is important, because it provides an opportunity to subject theoretical claims to empirical scrutiny. In particular, it provides an opportunity to assess whether the criteria for online forms of cognitive (and epistemic) extension can be satisfied by deliberate attempts at cognitive (and epistemic) systems engineering.

The paper has a second objective. In this case, the aim is to broaden the scope of theoretical debates pertaining to the possibility of Internet-extended knowledge. Many of these debates center on the nature of our interactions and engagements with systems such as Google Search (e.g., Schwengerer, 2021) and Wikipedia (Ludwig, 2015; Heersmink and Sutton, 2020). This focus is no doubt understandable given the popularity of these particular online systems, but the focus risks overlooking the different ways in which our bio-mental machinery might be interfaced to the online realm. The application described in the present paper is intended to illuminate some of these alternative technological paths to Internet-extended knowledge.

Before proceeding, it should be noted that there are (at least) two ostensibly distinct routes to extended knowledge (see Bjerring and Pedersen, 2014; Smart, 2018). One such route stems from a consideration of arguments for the extended mind and appeals to a state- or belief-based conception of knowledge. The other route focuses on the notion of extended cognition and emphasizes the role of extended cognitive processes in producing knowledge. The extent to which these two paths terminate in distinct forms of extended knowledge remains unclear, although, in the present paper, I will be focusing on the former route to extended knowledge. That is to say, I will be considering Internet-extended knowledge from the standpoint of claims about the possibility of extended mental states, such as states of dispositional belief.

## 2. HoloArt

For the most part, discussions of Internet-extended knowledge tend to focus on the forms of interaction and engagement enabled by a conventional Web browser. Some notable examples include the use of a Web browser to look things up on Wikipedia (e.g., Ludwig, 2015) or to perform a Google Search (e.g., Schwengerer, 2021). These forms of interaction are clearly important when it comes to understanding the epistemic impact of the Internet, but they do not exhaust the ways in which the online environment might be incorporated into epistemic routines. The case to be introduced here is intended to exemplify an alternative means of interacting with the online environment—one that exploits recent advances in mixed reality and cloud-based computing technology. This is what I will call the HoloArt case.

**Figure 1:**
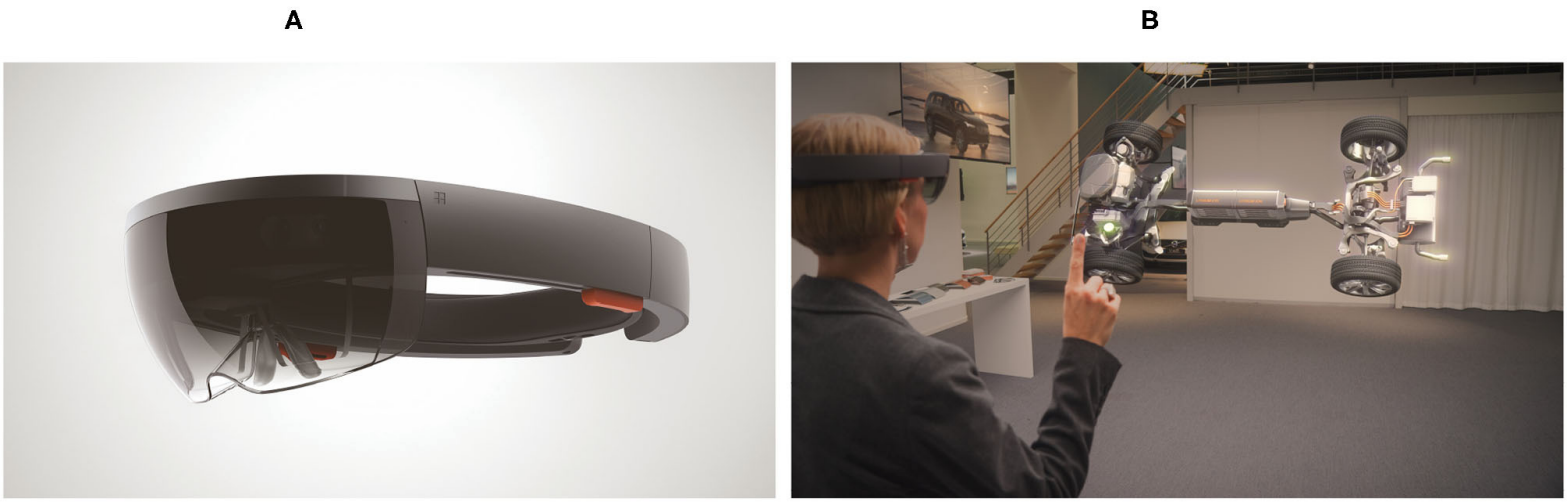
The Microsoft HoloLens. **(A)** The Microsoft HoloLens device. **(B)** An example of a HoloLens application. In this case, the HoloLens device enables a human user to visualize and interact with a virtual three-dimensional object (Used with permission from Microsoft).

The HoloArt case is based around the use of the Microsoft HoloLens, which is a head-mounted mobile computing device that allows a human user to interact with one or more virtual objects that are rendered in the local (physical) environment of the user (see Figure 1). Unlike immersive virtual reality headsets, such as the HTC Vive or the Oculus Rift, the HoloLens is a *mixed reality* device. This means that virtual objects—referred to as holograms—can be used to add information to the real-world (physical) environment of the human user. In the HoloArt case, we will be dealing with two-dimensional holograms that act as virtual annotations of a real-world physical object, namely, a painting. For most applications, however, holograms are rendered as three-dimensional objects that can be viewed from multiple angles. User interaction with the holograms is supported by a so-called Gesture, Gaze, and Voice (GGV) interface. This enables users to select virtual objects simply by looking at them (the Gaze component). Virtual objects can then be manipulated by performing certain hand gestures (the Gesture component), such as an air tap gesture (the HoloLens equivalent of a mouse click). Finally, the user can exploit the voice recognition capabilities of the HoloLens to invoke procedures or interact with virtual objects using simple voice commands (the Voice component). Such commands can, in some cases, serve as a substitute for hand gestures.

**Figure 2:**
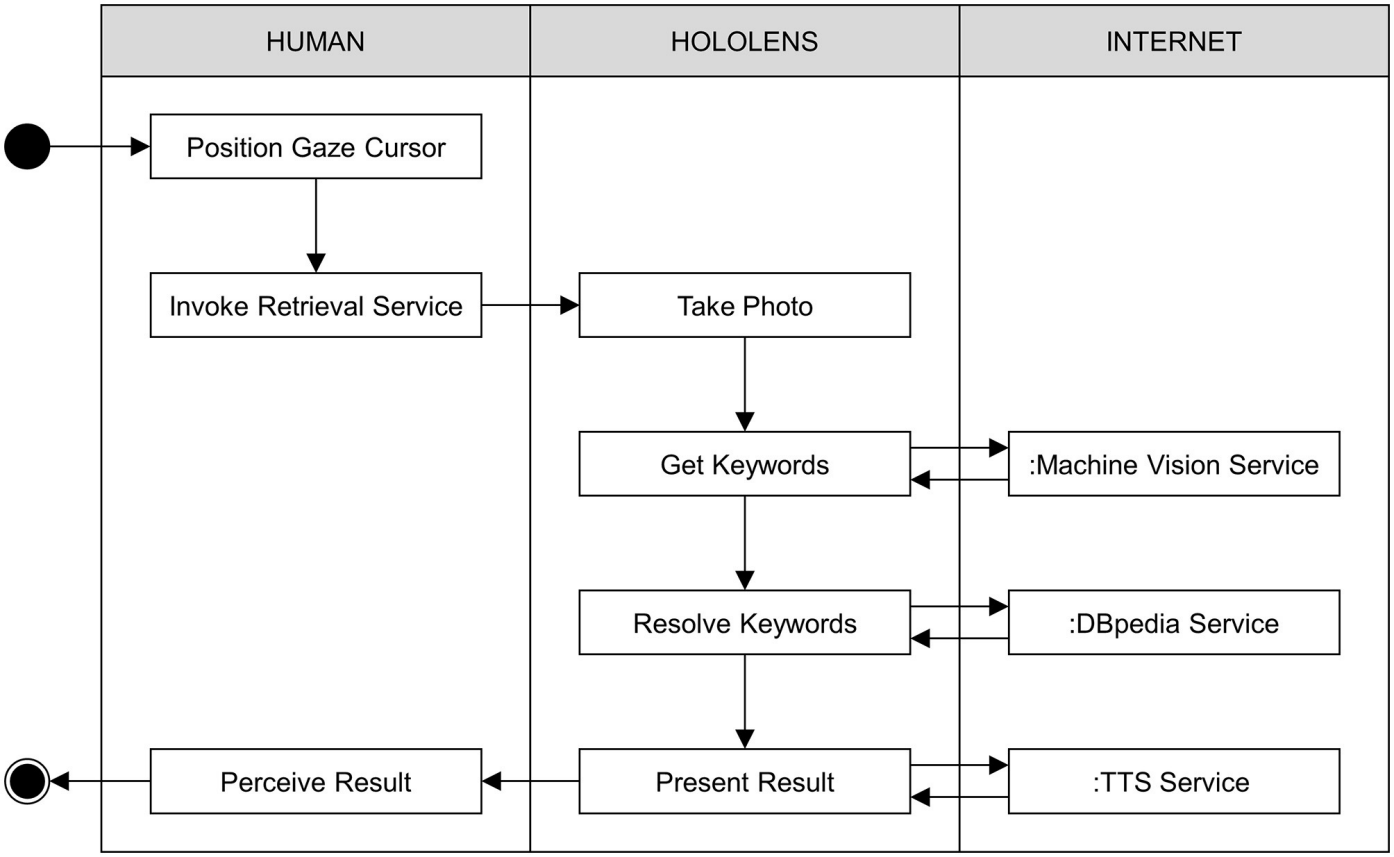
Swim lane diagram showing the distribution of activities between the human user, the HoloLens device, and online services in the case of the HoloArt app. Together, the individual steps of this routine comprise the painting recognition process.

As with many mobile computing devices (e.g., a smartphone), the functionality of the HoloLens is partly determined by the applications (or apps) that are installed on the device. (Similar to a smartphone, the HoloLens is equipped with a wireless connection, so individual apps can connect to the Internet or Web for the purpose of downloading online content or invoking online services.) The HoloArt case exploits the functionality of a real-world app, called the HoloArt app^1^. The goal of this app is to provide information about paintings, such as those that might be found in an art gallery. In particular, the HoloArt app aims to identify a painting that is within the field of view of a human user. It then returns information about the title of the painting and the artist responsible for the painting. Figure 2 illustrates the structure of the computational routine that implements this process. For the sake of convenience, I will refer to this process as the *painting recognition process*.

**Figure 3:**
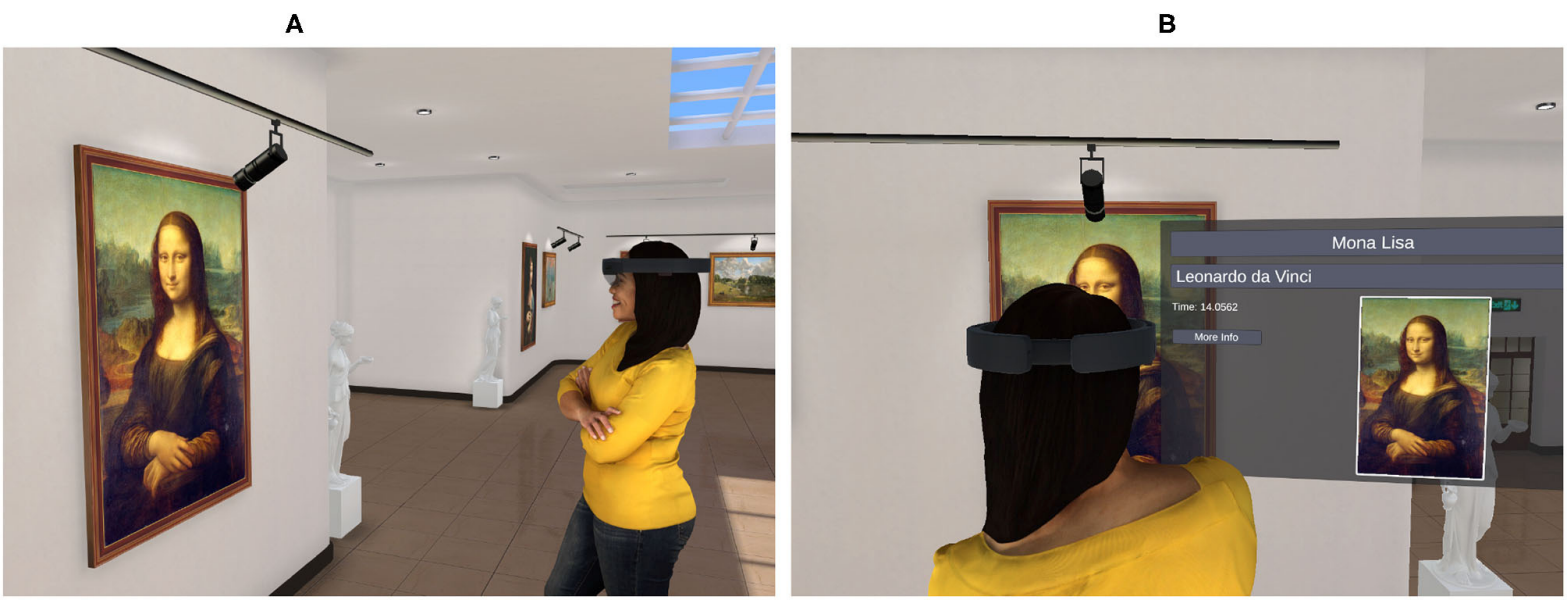
The HoloArt application. **(A)** The user triggers the painting recognition process by using a voice command or air tap gesture. **(B)** The result of the painting recognition process (if successful) is rendered as a holographic display panel that is spatially anchored to the location of the painting. A virtual copy of the painting is included in the display panel for verification purposes.

The basic functionality of the HoloArt app is as follows: In order to retrieve information about a painting, a user first positions a gaze cursor over the painting. (This can be achieved by simply looking at the painting.) The user then invokes the painting recognition process by performing a hand gesture (specifically, an air tap gesture) or by issuing a simple voice command (i.e., “IDENTIFY”). When these commands are detected by the HoloArt app, a photo of the current field of view is taken by the forward-facing camera of the HoloLens device (also known as the field camera). This photo is then posted to an online machine vision service, hosted by the Google Cloud platform^2^. The machine vision service analyses the uploaded photo and generates a list of keywords describing the photo^3^. These keywords are then returned to the HoloArt app. For each keyword, the HoloArt app connects to another online service, called the DBpedia service^4^. This service provides access to a machine-readable version of Wikipedia called DBpedia (e.g., Auer et al., 2007). By querying this service, the HoloArt app can check whether any of the keywords returned by the machine vision service correspond to the name of an entity described by Wikipedia. It can also check the properties of this entity, such as where (on Wikipedia) the entity is described, who created the entity, and whether the entity has a pictorial representation. To retrieve this information, the HoloArt app constructs a query for each of the keywords returned by the machine vision service. It then posts this query to the DBpedia service. If the query fails, the DBpedia service will return a null result. In this case, the HoloArt app tries the next keyword, continuing in this fashion until there are no more keywords remaining (for performance reasons, only the top five keywords are evaluated). If none of the keywords can be matched to anything on Wikipedia, the painting recognition process terminates in failure. The HoloArt app signals this failure with an auditory prompt (i.e., “COULD NOT IDENTIFY”). If one of the queries is successful, the DBpedia service returns the title of the painting and the name of the artist responsible for the painting. In this case, the HoloArt app generates a holographic display panel containing the returned information. This is rendered adjacent to whatever (real-world) painting the user was looking at when the painting recognition process was invoked (see Figure 3)^5^. In addition to the display panel, the HoloArt app exploits a Text-To-Speech (TTS) service (hosted by the Google Cloud platform), which provides the user with auditory feedback about the result of the painting recognition process. If, for example, a user is looking at the *Mona Lisa*, the HoloArt app will generate the following (speech) output: “MONA LISA BY LEONARDO DA VINCI”^6^.

The HoloArt app is intended to highlight the way in which contemporary digital technologies might be used to support extended knowledge. As should be clear from the foregoing characterization of the painting recognition process, these technologies are a somewhat mixed bunch. Firstly, we have the HoloLens device, which generates virtual objects (e.g., the display panel) in response to user actions. These virtual objects (or holograms) function as a sort of virtual post-it note that provides access to information about real-world objects. In effect, the HoloLens device is helping to enrich the real-world environment by annotating it in various ways. In the HoloArt case, these annotations are intended to provide access to *factual* information about a specific class of objects, namely, paintings, and they therefore provide the basis for what we might call factual or semantic knowledge^7^. There is, however, no reason why the HoloLens could not be used to add other kinds of information to the local environment of a user. Imagine, for example, a state-of-affairs in which a human user was guided through the performance of a complex task via the addition of virtual cues and affordances that were overlaid onto a variety of task-specific objects. Here, the holograms might be used to guide behavior in a manner that is perhaps more reminiscent of procedural knowledge.

In addition to the HoloLens, the HoloArt app relies on a number of online services. The first of these services is the machine vision service that is hosted by the Google Cloud platform. The use of this service highlights the way in which cloud-based Artificial Intelligence (AI) capabilities (in this case, a capacity for visual analysis) can be incorporated into epistemically-relevant routines.

**Figure 4:**
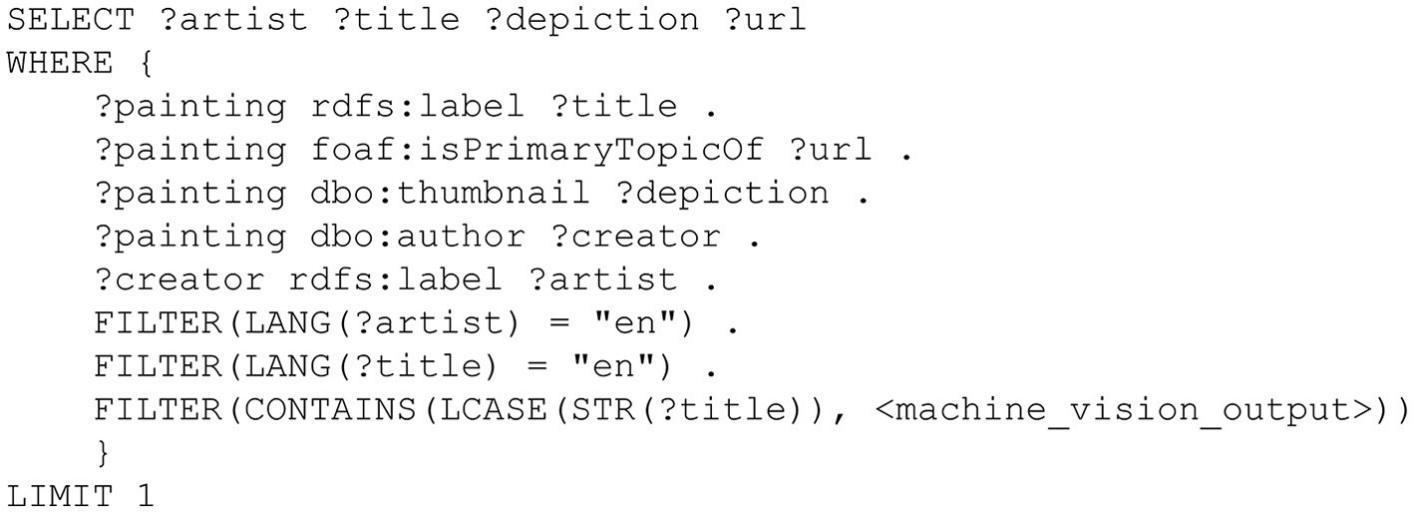
An example query used in the context of the HoloArt app. This query executes against a machine-readable version of Wikipedia, called DBpedia. The query can be executed against DBpedia (http://dbpedia.org/sparql) by replacing the term <machine_vision_output> with the title of a painting (Note the title should be in lower case and enclosed by quotation marks, e.g., “oath of the horatii”).

A second service exploited by the HoloArt app is the DBpedia service. This service supports the evaluation of the keywords generated by the machine vision service. As noted above, DBpedia is a machine-readable version of Wikipedia. It is, in effect, a structured repository of at least some of the information that is available via the Wikipedia system. Within DBpedia, information is represented in the form of a graph structure that loosely resembles some network-based models of human semantic memory (Collins and Quillian, 1969). This is what enables the HoloArt app to detect the presence of painting-related information in the list of keywords generated by the machine vision service. For each keyword, the HoloArt app constructs a query similar to that depicted in Figure 4 (for each query, the term  < machine_vision_output> is substituted with one of the keywords returned by the machine vision service). This query detects whether the keyword is a label (rdfs:label) that applies to a resource (?painting) that has a creator (dbo:author) and a depiction (dbo:thumbnail). If the query succeeds, then the variables specified in the SELECT clause (i.e., ?artist ?title ?depiction ?url) will be bound to values. These are the values that are returned to the HoloArt app, where they are used to generate the visual (display panel) and auditory (speech) outputs of the painting recognition process.

Having described the functionality of the HoloArt app, we are now in a position to consider the HoloArt case. The case features two protagonists: Gustav and Frida, with Gustav serving as the counterpart to Otto and Frida serving as the counterpart to Inga in the original Otto case^8^. Both Gustav and Frida find themselves in an art gallery. Gustav is equipped with a HoloLens, and he uses the HoloArt app while browsing the gallery's collection of paintings. If we ask Gustav about the details of a particular painting, he performs an air tap gesture and responds by telling us the name of the artist and the title of the painting. Unlike Gustav, Frida is not wearing a HoloLens. Frida has some basic knowledge about the paintings in the art gallery, such that if we ask her about a particular painting, she will respond by telling us the name of the artist and the title of the painting. For the purposes of the thought experiment, we can assume that the epistemic responses of Gustav and Frida are identical, such that if Gustav fails to recognize a painting, then Frida will also fail to recognize the painting. Similarly, if Gustav succeeds in recognizing a painting, then Frida will also succeed in recognizing the painting.

Given the similarity of Frida and Gustav's epistemic performances, we might be inclined to evaluate their epistemic standing in similar ways. After all, if we credit Frida with knowledge about a particular painting, then why demur from the idea that Gustav also possesses this knowledge? There is, of course, an important difference between Frida and Gustav. Gustav's epistemic responses are informed by a world-involving informational circuit—one that extends beyond his biological/organismic boundaries to include the HoloLens device and a number of distally-located (online) computational services. This is not the case with Frida, who, we may suppose, is relying on her brain-based neural circuits to retrieve painting-related information. Despite this difference regarding the mechanistic realization of the two retrieval-related routines, it is difficult to see why Frida and Gustav ought to be treated differently as regards matters of epistemic standing. Providing the behavior of the two protagonists is sufficiently similar, then perhaps they both ought to be admitted to the same epistemic club: If Frida knows about the paintings in the gallery, then it seems we ought to say the same for Gustav. Frida's knowledge is, of course, knowledge of the non-extended variety: her knowledge stems from the operation of circuits that are wholly contained within her biological/organismic boundaries. Gustav's knowledge, by contrast, is knowledge of the extended variety. In particular, Gustav's knowledge is rooted in the operation of circuits that extend beyond his organismic boundaries. (This is what marks the basic distinction between extended and non-extended knowledge.) What is more, the circuits responsible for Gustav's knowledge reach out into the online environment, incorporating the information processing activities of a variety of online services (e.g., the machine vision service). Accordingly, Gustav's knowledge is not just knowledge of the extended variety, it also qualifies as a form of Internet-extended knowledge^9^.

## 3. True Believers?

To what extent is it appropriate to say that Gustav is the beneficiary of (Internet) extended knowledge? In answering this question, we need to consider the extent to which Gustav meets the conditions associated with a philosophical account of knowledge. According to a classical account of knowledge, an agent *S* knows that *p* if, and only if, (i) *S* believes *p*, (ii) *S*'s belief that *p* is true, and (iii) *S*'s belief that *p* is justified (see Pritchard, 2009, p. 6). As noted, by Pritchard (2009, pp. 6–7), this account features an appeal to three conditions. Firstly, there is the doxastic condition. In order for *S* to know that *p, S* must believe *p*. Secondly, there is the factivity condition. In order for *S* to be credited with knowledge, then *S*'s belief that *p* must be true. Finally, we have what might be called a justification condition: *S*'s belief that *p* must be, in some sense, justified. Of these conditions, it is the justification condition that has proved to be the most contentious. Many epistemological accounts accept that knowledge is a form of true belief, but they vary with regard to what else must be added so as to elevate true belief to the status of knowledge. For process reliabilists, the added extra comes in the form of reliable processes (true beliefs must be produced by reliable processes); for virtue reliabilists, the missing ingredient is cognitive ability (true beliefs must stem from the exercise of cognitive ability); and for modal reliabilists, it is epistemic safety that is important (true beliefs should be formed in a manner such that they could not easily have been false).

For the time being, let us direct our attention to the doxastic condition and the factivity condition. The extent to which Gustav satisfies the doxastic condition will depend on whether it makes sense to regard Gustav as possessing painting-related beliefs. Given that this is the focus of the discussion in sections 4 and 5, let us postpone a discussion of that issue and direct our attention to the factivity condition. The factivity condition requires that an agent's beliefs are true. That is to say, whatever painting-related beliefs Gustav and Frida are deemed to possess, these beliefs must (at a minimum) be true in order to qualify as knowledge. If Frida should fail to correctly identify the title of a particular painting, then it doesn't seem appropriate to credit her with knowledge of that particular painting. Frida may, of course, insist that she knows the title of the painting. If, however, we ask her to identify the painting's title and she delivers an incorrect response, then it seems fair to conclude that she doesn't actually know the painting's title (and, by implication, that she did not know the painting's title even before she lodged a response to our question). For if she did know the painting's title, then she would have provided us with the correct answer. Similarly, in order for Gustav to be credited with knowledge, he must, at a minimum, be able to provide us with factually correct information about whatever paintings he is looking at. If we ask him to identify the title of a particular painting, and he delivers an incorrect response, then he clearly doesn't know what the title of the painting is. We might say that he possesses (extended) beliefs about the painting, but these beliefs won't amount to knowledge about the painting.

If we assume for a moment that Gustav simply reports the results of the painting recognition process, then the veracity of Gustav's responses will be tied to the reliability of the painting recognition process. If the painting recognition process correctly identifies every painting that Gustav is looking at, then Gustav will always report the correct result. Unfortunately, the painting recognition process is not 100% reliable. Some insight into the reliability of the painting recognition process is provided in Table 1. This table shows the results of the painting recognition process with a sample of 15 paintings. As is clear from this table, the painting recognition process sometimes delivers incorrect results. One point of failure for the painting recognition process is the machine vision service. Thus, if the machine vision service should misidentify the painting (perhaps because the target painting bears some visual similarity to another painting), then there is a good chance that the painting recognition process will yield an incorrect result. A second point of failure relates to the DBpedia service, which may return an incorrect result if multiple paintings have the same title. In Table 1, for example, we see that an incorrect result is returned for *The Kiss* by Gustav Klimt. The actual result returned by the HoloArt app is *The Kiss* by Francesco Hayez.

**Table 1:** Results of the painting recognition process with a sample of 15 paintings.

**Painting**	**Artist**	**Result**	**Time (s)**
Mona Lisa	Leonardo da Vinci	Correct	8.37
Napoleon Crossing the Alps	Jacques-Louis David	Correct	6.31
Death and Life	Gustav Klimt	Correct	5.30
Café Terrace at Night	Vincent van Gogh	Correct	5.46
Oath of the Horatii	Jacques-Louis David	Correct	6.40
Girl with a Pearl Earring	Johannes Vermeer	Correct	5.03
Portrait of Madame X	John Singer Sargent	Correct	5.88
Les Demoiselles d'Avignon	Pablo Picasso	Correct	4.63
San Giorgio Maggiore at Dusk	Claude Monet	Correct	6.10
The Kiss	Gustav Klimt	**Incorrect**	5.42
Nude Sitting on a Divan	Amedeo Modigliani	**Incorrect**	7.04
Ulysses Deriding Polyphemus	J. M. W. Turner	Correct	4.16
The Scream	Edvard Munch	Correct	6.35
Wivenhoe Park	John Constable	Correct	5.75

*Bold entries indicate incorrect responses. The “Time” column indicates retrieval latency in seconds*.

The HoloArt app includes a feature that is designed to deal with these error possibilities. Note that the query in Figure 4 includes a variable named ?depiction. If the query is successful, then this variable will contain a pointer to a graphic depiction (an online image) of the painting that is matched by the query. This depiction is included in the display panel that is generated by the HoloArt app. Thus, when the display panel is rendered, the HoloArt app downloads an online image of the painting and includes this in the display panel that is presented to the human user (see Figure 3). Note that this painting is *always* included in the display panel. If no online copy of the painting is available, then the ?depiction variable will not be bound to a value and the query will fail. In this case, the user will be presented with an auditory prompt signaling the failure of the painting recognition routine (i.e., “COULD NOT IDENTIFY”).

At this point, it should be clear that if the painting recognition process succeeds (i.e., delivers a non-null result), then the display panel will be presented, and Gustav will have an opportunity to check that the painting-related information is correct. In particular, Gustav can visually cross-check the virtual (holographic) copy of the painting (in the display panel) with the physical painting that is located in the real world. If these paintings match, then Gustav can be confident that the painting recognition process has correctly identified the (real-world) painting. If, however, the paintings do not match, then Gustav has an opportunity to reject the informational deliverances of the HoloArt app. The upshot is that it is difficult for Gustav to be led astray by the painting recognition process. In some cases, the HoloArt app may misidentify a painting and thus deliver an incorrect result, but Gustav is under no obligation to endorse this result. If Gustav is asked whether he knows the title of a specific painting, he can invoke the painting recognition process and perceive the result. If the virtual (i.e., holographic) painting matches the physical painting, then he will almost certainly deliver the correct response. If the paintings fail to match, however, then he can simply respond by saying that he doesn't know.

For the sake of convenience, let us refer to this verification process—the process of visually comparing the holographic painting with the real-world painting—as the *endorsement step*. The endorsement step ensures the veracity of Gustav's beliefs. It is, in short, a way of making any errors in the painting recognition process visible to Gustav. At a general level, the painting recognition process can return three types of result: it can correctly identify the target painting and thus deliver a *correct result*; it can fail to correctly identify the target painting and thus deliver an *incorrect result*; or it can simply fail to deliver any sort of result, thereby yielding a *null result*. Of these results, it is only the correct results that form the basis for Gustav's painting-related beliefs. Gustav does not believe null results, for null results do not provide any information about a painting. Similarly, Gustav cannot be said to believe incorrect results, for these results will be rejected following the performance of the endorsement step. For both null results and incorrect results, Gustav will respond in a similar way to epistemic challenges: he will simply say he doesn't know. The only situation in which Gustav will furnish us with painting-related information is when the holographic painting matches the real-world painting, and these are the situations in which the painting recognition process has correctly identified the target painting.

The upshot is that Gustav is a highly reliable source of painting-related information. By itself, the painting recognition process is not 100% reliable, but when we consider the reliability of the larger epistemic system comprising Gustav, the HoloLens, and the remotely-situated online services, then we do confront a system that is highly reliable, at least in ecologically normal circumstances. This is not to say that there is absolutely no way for Gustav to be led astray by his nexus of technological resources. It is perhaps possible for the reliability of the larger epistemic system to be compromised in some way—some determined hacker, for example, might be able to subvert the workings of the painting recognition process. On the whole, however, these situations are unlikely to arise, for the nature of the painting recognition process makes it very difficult for misleading information to delivered to the human user^10^. Accordingly, the painting recognition process might be seen to satisfy the demands of a safety-related (modal reliabilist) approach to knowledge: Inasmuch as Gustav possesses painting-related beliefs, then these beliefs are not ones that could have easily been false (see Pritchard, 2009, p. 34). The upshot is that the HoloArt case establishes a sensible point of contact with recent attempts to examine extended knowledge from a safety-based epistemological perspective (e.g., Hirvelä, 2020).

In addition to safety-based or modal reliabilistic approaches to extended knowledge, the notion of extended knowledge has also been examined from a virtue epistemological perspective (Pritchard, 2010, 2018). Of particular importance is an epistemological position known as virtue reliabilism. A key feature of virtue reliabilistic accounts is the emphasis they place on cognitive abilities in determining the truth of an individual's beliefs. In particular, virtue reliabilists insist that knowledge is the product of cognitive ability. “[T]o say that someone knows,” Greco (2003, p. 111) suggests, “is to say that his believing the truth can be credited to him. It is to say that the person got things right owing to his own abilities, efforts, and actions, rather than owing to dumb luck, or blind chance, or something else.” This is what Pritchard (2010) refers to as the ability intuition:

A true belief, no matter what else of epistemic relevance can be offered in its favor (e.g., that it is safe, sensitive, backed by reasons, epistemically blameless, and so on), will not count as a case of knowledge if it is not the product of cognitive ability. Call this the ability intuition. (Pritchard, 2010, p. 134)

The upshot is an account of extended knowledge that draws attention to the role of cognitive ability in securing positive epistemic standing. From a virtue reliabilistic perspective, Gustav's painting-related beliefs ought to be true as the result of the exercise of cognitive abilities; in particular, the beliefs ought to be true as the result of cognitive abilities that are ascribed to Gustav (or whichever entity/agent is the target of knowledge attribution). The endorsement step provides us with a means of satisfying this condition. To help us see this, note that while the painting recognition process clearly plays a role in determining the *content* of Gustav's beliefs, the truth of these beliefs is not wholly determined by the painting recognition process. If we ask ourselves *why* it is that Gustav believes the true (or why Gustav provides us with a *correct* response to painting-related questions), our answer probably ought to reference the role that Gustav plays in verifying the results of the painting recognition process. It is, after all, Gustav (the biological agent) who is performing the endorsement step, and the endorsement step plays a role in determining whether or not Gustav's subsequent behavior (e.g., his verbal responses to painting-related questions) will be coordinated with respect to the factive structure of reality. In this sense, Gustav is at least partly responsible for the way that online information influences his thoughts and actions^11^. If the painting recognition process should prove unreliable, and Gustav should also fail to implement the endorsement step, then at least some of Gustav's responses to art-related questions will be incorrect. If we then ask ourselves who (or what) is responsible for these incorrect responses, it seems that some of the blame must lie with Gustav. After all, if Gustav had performed the endorsement step, then he wouldn't have furnished us with these incorrect responses.

The endorsement step thus provides a means of satisfying the constraints imposed by the ability intuition. By performing the endorsement step, Gustav influences the extent to which his thoughts and actions are aligned with certain facts about the world^12^. This alignment occurs as the result of a visual comparison process that reflects the exercise of visuo-cognitive abilities. Furthermore, there seems little reason to reject the idea that these abilities ought to be ascribed to the entity we recognize as Gustav. The upshot is that Gustav satisfies the ability intuition: Gustav believes the true as the result of the exercise of cognitive abilities that are properly ascribed to him^13^.

We thus have at least two ways of thinking about extended knowledge: we can either look at extended knowledge from the perspective of a modal reliabilistic or virtue reliabilistic approach to knowledge. Epistemologists will, of course, vary as to which of these accounts (if any) ought to serve as the basis for our understanding of extended knowledge. For present purposes, however, I hope to have shown that neither of these epistemological accounts ought to be seen as incompatible with the possibility of extended knowledge in the HoloArt case. While claims about extended knowledge have typically been approached from a virtue reliabilistic perspective, there is, I think, no reason why the nature of the human–technology interactions associated with the HoloArt app ought to be seen as inconsistent or incompatible with alternative ways of approaching the notion of extended knowledge.

## 4. Trust and Glue

Issues of reliability are clearly important when it comes to claims about extended knowledge (or indeed non-extended knowledge). There is, however, more to extended knowledge than a simple capacity to coordinate one's (verbal and behavioral) responses with respect to the factive structure of reality. In designing systems to support extended knowledge, we need to consider a set of constraints pertaining to the functional operation of the circuits that support attributions of belief and knowledge. In particular, a world-involving circuit (such as the painting recognition process) should operate so as to respect the constraints associated with the folk psychological strategy of explaining/predicting behavior via the ascription of mental states. A useful comparison here is with Frida's bio-memory circuits: Inasmuch as we ascribe beliefs and knowledge to Frida, then it seems that these folk psychological characterizations must be tied to the operation of Frida's bio-memory circuits. Accordingly, if Gustav is to be characterized in the same way as Frida, then there must be some degree of functional similarity between the circuits that sustain the relevant (epistemic) behaviors of the two protagonists.

The appeal to functional similarity is both valid and important, but it needs to be understood in the right light. Crucially, the relevant form of similarity does *not* relate to the way in which some body of information is retrieved from a source location. There is, for example, no need for the individual steps of the painting recognition process to correspond to the steps implemented by Frida's bio-memory process. Instead, what we are looking for is a state-of-affairs in which Gustav's world-involving circuit influences his behavior in more or less the same way as Frida's bio-memory circuit influences her behavior (where the relevant behaviors are those supporting the ascription of beliefs and knowledge to the two protagonists). The similarity thus comes from the way in which each circuit is apt to fulfill the functional role that we typically associate with certain mental states (such as the state of believing that painting *P* is painted by artist *A*).

Our task, then, is to compare Gustav's world-involving information retrieval circuit with Frida's brain-based information retrieval circuit. We are, however, not particularly interested in the fine-grained (implementation-level) details of the mechanisms that realize the two information retrieval processes. Instead, we are interested in a more abstract set of properties concerning the so-called “functional poise” (see Clark, 2010b) of the retrieved information (i.e., the extent to which the retrieved information is poised to guide thought and action in the manner we expect of information retrieved from bio-memory).

Some insight into the nature of these properties is provided by Clark and Chalmers (1998). In particular, Clark and Chalmers (1998) identify a set of criteria that are intended to guide our intuitions as to when some non-biological resource (e.g., Otto's notebook) ought to be seen as a candidate for inclusion in an individual's cognitive system. These criteria are what have come to be known as the trust+glue criteria. Clark (2010b, p. 46) recounts these criteria as follows:

1. **Availability Criterion:** That the resource be reliably available and typically invoked (Otto always carries the notebook and won't answer that he “doesn't know” until after he has consulted it).
2. **Trust Criterion:** That any information thus retrieved be more or less automatically endorsed. It should not usually be subject to critical scrutiny (unlike the opinions of other people, for example). It should be deemed about as trustworthy as something retrieved clearly from biological memory.
3. **Accessibility Criterion:** That information contained in the resource should be easily accessible as and when required.

According to Clark and Chalmers (1998), Otto's notebook satisfies these criteria, and thus the notebook ought to be regarded as part of the collection of physical resources that constitutes Otto's mind. Things are a little more complicated when we seek to apply these criteria to the HoloArt case. For a start, the HoloArt case features a multiplicity of resources (e.g., the HoloLens device and multiple server computers), and it is not clear which of these resources are targeted by the trust+glue criteria. It is also worth noting that the wording of the accessibility criterion is not particularly appropriate to the HoloArt case. In particular, there is no sense in which the informational deliverances of the painting recognition process are “contained in” the HoloLens; rather, the HoloLens provides a means of accessing information that is located elsewhere (see section 5, for more on this).

For present purposes, let us set aside these issues and consider whether Gustav stands any chance of satisfying the trust+glue criteria. Given that we will consider the availability criterion in section 5, let us begin by examining the trust criterion. According to this criterion, the informational deliverances of the HoloLens ought to be “more or less automatically endorsed.” In one sense, Gustav looks ill-equipped to meet this criterion. After all, Gustav does *not* automatically endorse whatever information the HoloLens delivers. Instead, Gustav participates in an endorsement step to verify that the HoloLens has delivered the correct information (see section 3). From an epistemological perspective, the inclusion of this step looks to be important, for we do not want Gustav to be led astray by the painting recognition process. If something should go awry with the painting recognition process, then we want Gustav to be sensitive to this fact, and, if necessary, reject the informational deliverances of the HoloLens. The problem is that by including the endorsement step we seem to have inadvertently undermined the extent to which bio-external information is poised to play the functional role of a belief state. In particular, the endorsement step looks to be incompatible with the trust criterion: By introducing the endorsement step, it no longer seems appropriate to regard Gustav as *automatically* endorsing the informational outputs of the painting recognition process.

At this point, we confront something of a problem, for it is not particularly clear what it means for an agent to *automatically* endorse information from a bio-external source. Nor is it particularly clear why this appeal to automatic endorsement should be crucial for claims about the extended mind. The wording of the trust criterion suggests that a parallel is being drawn with biological memory—that we ought to endorse information in more or less the same way we endorse information retrieved from bio-memory. But, as some philosophers have noted, it is not clear whether this notion of automatic endorsement makes much sense when applied to biological memory. Michaelian (2012), for example, suggests that “…stored information is not automatically believed by the subject upon retrieval…. Some records stored in memory will not count as dispositional beliefs, since the subject does not tend to endorse them upon retrieval” (p. 1159).

We thus have two issues to resolve: the first relates to the purpose of the appeal to automatic endorsement in arguments for the extended mind; the second relates to whether the nature of the endorsement step (in the HoloArt case) invalidates claims of extended belief and knowledge.

As regards the first of these issues, I want to suggest that one of the reasons automatic endorsement is important is because it avoids a state-of-affairs whereby we ascribe beliefs to an agent and the agent then fails to act in accordance with those beliefs, perhaps because they have subjected some body of action-guiding information to critical scrutiny. Suppose we ascribe a set of dispositional beliefs to Otto based on the informational contents of his notebook. We then learn that Otto is somewhat circumspect about the informational deliverances of his notebook “device.” This revelation is important, for it opens the door to situations in which Otto's overt behavior might run counter to the patterns of behavior that are (in a folk psychological sense) explained by the ascription of notebook-based beliefs to Otto. Suppose we look at Otto's notebook and discover that it contains a statement about MOMA's location: “MOMA is located on 53rd Street.” Given the assumption that Otto will automatically endorse whatever information is written in his notebook, this statement provides us with some insight into Otto's future behavior: Thus, if Otto desires to go to MOMA, we can infer that he will go to 53rd Street. The result is that we are in a position to ascribe dispositional beliefs to Otto. In particular, the appeal to automatic endorsement enables us to treat the notebook encodings as yielding an explanatorily- and predictively-potent grasp over Otto's actual (and counterfactual) behavior. If Otto desires to go to MOMA, he will go to 53rd Street (and not 52nd Street), and the reason he goes to 53rd Street (and not 52nd Street) is because he desires to go to MOMA and he *believes* that MOMA is on 53rd Street.

This is all well and good. But now suppose that we learn that Otto is somewhat circumspect about the information written in his notebook. In this case, we have no guarantee that Otto will endorse the statement about MOMA being on 53rd Street. If Otto desires to go to MOMA, he will access the notebook and read the statement about MOMA's location. But there is no guarantee that he will then head off to 53rd Street. Upon reading the statement about 53rd Street, Otto may decide that the information is incorrect and go to 52nd Street. If so, it would clearly be inappropriate to credit Otto with the belief (let alone knowledge) that MOMA is on 53rd Street; for if Otto did, in fact, believe that MOMA was on 53rd Street, then he wouldn't have gone to 52nd Street—he would have gone to 53rd Street.

To my mind, this helps us understand *why* the notion of automatic endorsement is important when it comes to arguments for the extended mind. The appeal to automatic endorsement is intended to avoid a state-of-affairs in which we encounter a disconnect between the action-guiding role of some body of bio-external information and the actual patterns of behavior that stem from the processing of that information. This is clearly important when it comes to the ascription of dispositional beliefs: If Otto does not automatically endorse the informational deliverances of his notebook, then he might act in a way that is counter to the assumed action-guiding role of the notebook encodings. In such cases, it is (at best) unclear that we should treat the notebook encodings as providing us with any sort of folk psychological grip over Otto's actual and counterfactual behavior.

The question is whether this sort of constraint is being violated by the introduction of the endorsement step in the HoloArt case (this is where we move to the second issue regarding the compatibility of the endorsement step with claims about the extended mind). My sense is that there is no conflict here. If the painting recognition process succeeds in (correctly) identifying a painting, then Gustav will endorse the result of the painting recognition process. If, on the other hand, the painting recognition process does not succeed in (correctly) identifying a painting, then Gustav won't endorse the result of the painting recognition process. There is, I suggest, no real disconnect here between the mechanism responsible for the operation of the painting recognition process and the expression of behavior that warrants the ascription of mental states (e.g., states of dispositional belief) to an agent. If the painting recognition process *always* succeeds in identifying a particular painting (e.g., the *Oath of the Horatii*), then it seems reasonable to conclude that Gustav knows something about this painting. After all, whenever we present Gustav with this painting, and we then challenge him with questions, he always delivers the corrects answers.

To my mind, then, there is nothing problematic about the endorsement step vis-à-vis the trust criterion. The endorsement step is merely a way of “gating” the flow of information within a larger extended (world-involving) circuit, one that culminates in the expression of behaviors that are subsumable under familiar folk psychological kinds: kinds such as “Gustav believes that the *Oath of the Horatii* was painted by Jacques-Louis David.” The mere presence of this “gating” process does not materially alter our ability to ascribe beliefs to Gustav, any more than a similar form of “gating” process in the original Otto case would affect our ability to ascribe beliefs to Otto. Consider, for example, a variant of the Otto notebook case where Otto's notebook is populated by a mixture of crossed-out statements (e.g., MOMA is located on 52nd Street) and non-crossed-out statements (e.g., MOMA is located on 53rd Street). Call this the Crossed-Out Otto case. Providing we know that Otto will ignore all the crossed-out statements and automatically endorse the non-crossed-out statements, then is there nothing to prevent us from ascribing dispositional beliefs to Otto, just as we did in the original Otto case. The appeal to automatic endorsement ensures that an individual's thoughts and actions will be coordinated with respect to some body of bio-external information. But this is something we can have, even if the body of endorsed information is interspersed with information that is ignored, rejected, or disregarded. Just like the Crossed-Out Otto case, the HoloArt case presents us with a situation in which some information is ignored, while other information is accepted, and just like the Crossed-Out Otto case, there is no reason why we cannot continue to ascribe beliefs to an individual based on our understanding of how an individual responds to bio-external information. If Otto's endorsement of notebook-related content is limited to non-crossed-out statements, then such statements serve as the basis for belief ascriptions. Similarly, if Gustav's endorsement activities are limited to holographic paintings that match real-world paintings, then we know that the information presented in these situations will serve as the basis for belief ascription. All that remains is the need to know what information will be returned in respect of particular paintings. If the painting recognition routine always delivers a correct result for the *Oath of the Horatii*, then we are in a position to say that Gustav possesses (extended) beliefs about the *Oath of the Horatii*^14^.

For those who remain unconvinced about the compatibility of the endorsement step with extended mind criteria, there is another of thinking about the endorsement step. Consider that the endorsement step provides an opportunity for Gustav to check the results returned by each token instantiation of the painting recognition process. Over time, however, the endorsement step provides information about the general reliability of the painting recognition process. Suppose, for example, that the painting recognition process is 100% reliable: Every time the process is invoked, it always returns a correct result. In this situation, Gustav may simply come to accept the informational deliverances of the HoloLens to the point where he no longer bothers with the endorsement step. At this point, he may deem the delivery of a holographic version of the real-world painting to be unnecessary and use one of the settings of the HoloArt app to disable the presentation of the display panel. Gustav may also come to learn about the conditions that affect the reliability of the painting recognition process. Perhaps, for example, Gustav learns that he needs to be at a certain distance from the painting in order for the correct result (or perhaps any result) to be returned. Over time, Gustav may come to adjust his own behavior (e.g., standing the right distance from the painting) so as to maximize the reliability of his world-involving circuit and (by implication) his own epistemic standing.

The basic point here is that we do not need to think of the endorsement step as something that needs to be performed *every* time the painting recognition process is invoked. The endorsement step can also be seen to provide information about the general reliability of the painting recognition process, and thus the extent to which the endorsement step is actually required. Perhaps, then, we can see the endorsement step as part of the “developmental history” of an Internet-extended knower. On first using the HoloArt app, Gustav may be somewhat circumspect about the informational deliverances of the HoloLens. Over time, however, he simply comes to accept that the HoloArt app will always deliver the correct result, at which point he disables the presentation of the holographic display panel and relies on the synthetic speech outputs of the HoloArt app. This, of course, assumes that the HoloArt app functions in a reliable manner, although if the HoloArt app should prove utterly unreliable, then Gustav may decide that this particular form of bio-technological bonding is no longer worth pursuing.

Having discussed the trust criterion, let us now direct our attention to the accessibility criterion. In contrast to the trust criterion, the accessibility criterion looks to be relatively unproblematic. At the very least, the accessibility criterion appears no more problematic for the HoloArt case than it is for the Otto case. The painting recognition process can thus be invoked by performing a simple hand gesture or by uttering a voice command. These actions are arguably simpler than those associated with the retrieval of information from a paper-based notebook. In addition, the results of the painting recognition process are presented to the user in a form that is easy to understand. Users can thus rely on the informational contents of the holographic display panel, or they can attend to the speech outputs generated by the HoloArt app. Once again, these forms of information uptake and exploitation appear no more effortful, difficult, or time-consuming than those encountered in the Otto case.

In one sense, then, the accessibility criterion poses little in the way of a problem for the HoloArt case. Perhaps, however, we are overlooking an important feature of the accessibility criterion. Presumably, the primary purpose of the accessibility criterion is to ensure that bio-external information is suitably poised to influence thought and action in the manner we expect of information retrieved from bio-memory. The way in which information is retrieved and presented to a human user is clearly important here, but such issues probably play second fiddle to a much more important concern, namely, the *speed* with which some sort of request for information is able to exert a cognitive and behavioral impact on a human subject. What this means, in the context of the HoloArt case, is that the painting recognition process should complete within a certain timeframe. If too much time elapses between the retrieval request (e.g., the air tap gesture) and information delivery (e.g., the presentation of the holographic display panel), then our intuitions about Gustav's epistemic standing might be called into question.

The relevant contrast, here, is with information retrieved from bio-memory. Such information is typically retrieved quite quickly. This is not to say that the information is *immediately* available—sometimes we have to think for a moment before the desired information comes to mind. Nevertheless, in many cases, we are able to recall information without too much of a delay. The question, of course, is how much of a delay is acceptable? Suppose we ask Frida if she knows the title of a particular painting and instead of providing us with an answer she responds with a protracted silence. At what point do we conclude that Frida does not, in fact, know the answer to our question? A few seconds is probably fine. Thirty seconds might be pushing it. A few minutes is probably too much. Even if Frida was able to provide us with a correct answer at this point (and our patience was sufficient to ensure that we were still around to hear the answer), we might not feel it is appropriate to credit her with knowledge. In attributing knowledge to Frida, we assume that she will be able to respond to our epistemic challenges in an appropriate and timely manner. If Frida knows (in a dispositional sense) that Jacques-Louis David painted the *Oath of the Horatii*, then she should be able to retrieve, recall, or remember this information without too much difficulty. That is to say, in crediting Frida with (dispositional) knowledge, we expect the mechanisms that sustain her (occurrent) epistemic performances to execute (and complete) within a certain timeframe.

How, then, does Gustav fare with respect to this temporally-inflected reading of the accessibility criterion? For the paintings listed in Table 1, it takes about 6 s (mean: 5.87 s; standard deviation: 1.05 s) for the painting recognition process to present results to the human user. For the kind of knowledge we are talking about here (i.e., semantic knowledge), I suspect this latency lies at the upper bound of what would be deemed acceptable for knowledge attribution. In general, faster response times are likely to be more compatible with knowledge attribution, such that beyond a certain point (e.g., 10 s) observers are less likely to credit someone with knowledge (regardless of the veracity of their responses)^15^. I suspect the same may be true of an individual's *subjective* responses to the operation of world-involving circuits. Thus, when information retrieval latencies are kept within certain temporal bounds, an individual may be more likely to report epistemic feelings (e.g., the feeling of knowing) that are consistent with the cognitive incorporation of bio-external information (see Clark, 2007, for more on this).

These are, of course, no more than hypotheses at this point; nevertheless, the status of the HoloArt app as a real-world application opens the door to future empirical work that seeks to evaluate people's folk psychological responses (both subjective and social) to situations involving the HoloArt app. The app could thus be used in real-world situations (e.g., in an actual art gallery) to assess the relationship between retrieval latency (as well as other application-specific performance variables) and the social/subjective evaluation of an individual's epistemic status.

## 5. The Occasional Knower

We have noted some of the similarities between Gustav and Frida when it comes to their ability to identify paintings. At some point, however, the tour of the art gallery must come to an end. What happens then? In all likelihood, Gustav will remove the HoloLens before he departs the art gallery. At this point, we seem to encounter a significant shift in Gustav's epistemic standing. While Gustav was wearing the HoloLens, it might have seemed appropriate to credit him with dispositional beliefs and knowledge about the paintings adorning the gallery walls. After all, whenever we ask Gustav about a particular painting, we receive a correct response to our query. This, we may suppose, justifies the attribution of knowledge to Gustav, at least for the time he is wearing the HoloLens. Arguably, however, part of the reason we attribute *dispositional* beliefs and knowledge to an agent is to gain a predictive toehold over that agent's *future* behavior. If we say that Gustav and Frida know about the paintings in the art gallery, then we probably expect them to recognize the paintings even when they are *not* in the art gallery. This looks to be fine for Frida. Suppose we meet with Frida the day after our visit to the art gallery. We present Frida with a picture of a painting from the gallery's collection and ask her to identify the painting's title. Inasmuch as Frida was able to identify the painting the day before (when she was located in the art gallery), she should still be able to identify the painting at our subsequent meeting (when she is not located in the art gallery). Accordingly, Frida's (non-extended) knowledge exhibits a degree of what we might call *location invariance*—we do not expect Frida's epistemic performances to be overly affected by moment-to-moment shifts in her spatial (or geographical) location.

Now let us re-run the case with Gustav: We meet with Gustav the day after the gallery visit. We present him with a picture of a (previously encountered) painting and ask him to identify the painting. Inasmuch as Gustav is no longer wearing the HoloLens, he may be in some trouble here. He may say that he doesn't know or that he can't remember. Given the nature of the knowledge in play here (i.e., semantic knowledge), such performances are apt to strike us as somewhat odd, and they may make us wonder whether it was appropriate to credit Gustav with knowledge in the first place. If Gustav is deemed to possess dispositional knowledge of the paintings in the gallery, then shouldn't he be able to identify those paintings when he is not in the gallery? If Gustav can only respond to our epistemic challenges in specific situations—e.g., while he is located in the art gallery—then it can't be the case that Gustav possesses dispositional knowledge. For if Gustav did possess dispositional knowledge (of the sort possessed by Frida), then he ought to be able to respond to our epistemic challenges in roughly the same kinds of situations in which Frida is herself challenged.

The problem, here, is the temporary nature of Gustav's epistemic contact with the online environment. Gustav only appears to possess knowledge of the paintings when the HoloLens is worn and the HoloArt app is functioning correctly. The HoloLens is, however, a temporary fixture. As soon as Gustav removes the HoloLens, he no longer has an ability to identify artworks. Inasmuch as Gustav is credited with knowledge, then this knowledge appears to come and go. This contrasts with the nature of Frida's knowledge, which appears much more enduring. Frida is, of course, relying on her bio-memory circuits, and these circuits are internal to Frida. Accordingly, the circuits (and the epistemic performances they support) tend to be associated with Frida: wherever Frida goes the bio-memory circuits are sure to follow. This is not the case for the world-involving circuits that sustain Gustav's epistemic performances. Such circuits are immediately lost the moment the HoloLens is removed. They are also lost in a range of other situations, such as if the HoloLens battery should die, the WiFi connection should be lost, or the HoloArt app should unexpectedly crash. Such forms of fragility and impermanence may have a significant impact on Gustav's epistemic credentials. If we can no longer rely on Gustav to provide us with correct answers to questions on multiple occasions, then perhaps he no longer ought to be credited with knowledge. As a means of reinforcing this particular point, consider how we might regard an agent who is able to answer questions about the paintings in an art gallery simply by reading the (real-world) labels printed below each painting. This agent, we may suppose, is in a similar position to Gustav. Just like Gustav, this agent will be able to identify paintings while they are in the art gallery. And just like Gustav, they will no longer be able to identify paintings once they are outside the art gallery. Inasmuch as we fail to regard this label-reading agent as a genuine knower, is there any reason to regard Gustav any differently?

Relative to debates about the extended mind, the kind of problem we are encountering here (e.g., the location-specific nature of epistemic performances) arises as a result of the failure to satisfy the availability criterion (see section 4). According to this criterion, a bio-external resource (e.g., the HoloLens device) ought to be “reliably available and typically invoked” (Clark, 2010b). But neither of these conditions are likely to be met in the HoloArt case. For much of the time, Gustav will not be wearing the HoloLens, so the informational circuits that sustain his epistemic responses will not be available to him. Nor do we have any reason to assume that Gustav will *typically* resort to the use of the HoloLens whenever we ask him whether he knows about a particular painting.

We could, of course, address these problems by modifying the HoloArt case. Perhaps, for example, we could envisage a state-of-affairs in which Gustav is equipped with a futuristic retinal display device (e.g., a contact lens) that provides the same functionality as that observed in the HoloArt case. In this situation, Gustav may have more or less constant access to information about paintings that lie within his field of view. That is to say, whenever Gustav looks at a painting, information about the painting will be displayed in the form of a Terminator-style augmented reality display, and he will thus always have access to painting-related information (as well as perhaps a great deal of other information). Inasmuch as this satisfies the demands of the availability criterion, then it would seem that technological innovation (e.g., advances in wearable technology) may hold the key to extended knowledge.

There is, however, another possibility. Before we accept the need for futuristic technology, it is worth re-examining the availability criterion in the specific context of claims about *Internet*-extended knowledge. This is important, for the availability criterion was originally formulated in the context of the Otto case. This case bears some similarity to the HoloArt case, but there are also some important differences. Consider, for example, the relationship between Otto's notebook and the informational encodings contained within the notebook. The information that Otto accesses is written on the pages of the notebook, so there is a reliable association between the notebook (the extraorganismic resource) and the information that Otto exploits. Accordingly, if Otto is to be credited with knowledge about (e.g.,) MOMA's location, he better be accompanied by his notebook.

Now let us turn our attention to Gustav. Because Gustav uses the HoloLens to access the information he needs, we might be inclined to think of the HoloLens in the same way we think of Otto's notebook. This, however, is a mistake. As was noted in section 4, the information that Gustav needs is *not* “contained in” the HoloLens; rather, the HoloLens is being used to mediate Gustav's access to information that is retrieved from the online realm. This is important, for if Gustav should have some other means of accessing the online environment, then it is not clear that Gustav needs to invoke the services of the HoloLens *every* time he requires access to painting-related information. Relative to issues of extended knowledge, what seems to be important here is not so much the continuous presence of some particular bio-external *resource* (e.g., a HoloLens device), but rather the reliable presence of the *information* that sustains epistemically-relevant performances^16^. In the HoloArt case, the HoloLens device is certainly one of the means by which Gustav can access epistemically-relevant information, but it need not be the only way that Gustav can access this information. Gustav may rely on the HoloArt app when he is wearing the HoloLens, while at other times he may rely on a smartphone app to provide him with more or less the same functionality^17^. The fact that these world-involving informational circuits are constituted by a multiplicity of different devices does not seem particularly important to Gustav's status as an *Internet*-extended knower. What matters is the fact that each of these devices is able to preserve the functional poise of online information, thereby enabling Gustav's thoughts and actions to be influenced in a manner that is consistent with the folk psychological strategy of explaining/predicting behavior via the ascription of mental states. Providing they do this, then there is no need for the HoloLens device to be continuously available to Gustav. Nor is there any need for Gustav to invoke the services of the HoloLens *every* time he needs to access painting-related information. As long as Gustav is able to exploit a multiplicity of different devices to preserve his informational contact with the online environment, then there is no need for Gustav's epistemic credentials to evaporate the moment he removes the HoloLens. Gustav will thus remain an Internet-extended knower just so long as he retains the right sort of informational contact with the online environment. In all likelihood, this is something that can be achieved with today's technologies, and there is thus no need for Gustav to await the arrival of some “futuristic” form of cognitive technology, such as a retinal display device, a Neuralink chip, or some other technological resource that can be permanently pinned to the biological skinbag^18^.

## 6. Conclusion

The present paper describes a putative case of Internet-extended knowledge based around a real-world application, called the HoloArt app. The primary purpose of the HoloArt app is to show how contemporary digital technologies might be used to support the practical effort to engineer extended epistemic systems—systems that deliver knowledge by providing circuits that are apt for cognitive incorporation. In the present case, these circuits exploit the functionality of a mixed reality device in the form of the Microsoft HoloLens. They also reach out into the Internet, drawing on systems and services that are emblematic of recent advances in cloud computing (i.e., the Google Cloud platform), machine intelligence (i.e., the machine vision service), and data science (i.e., the DBpedia service). In part, the choice of these technologies was motivated by the effort to address issues and concerns that have arisen in respect of the philosophical notion of extended knowledge. There was, however, an additional motivation for the HoloArt app: the app serves as an important reminder of the different ways that our bio-mental machinery might be interfaced to the online realm. Thus, just because one form of interaction and engagement with the Internet fails to meet the conditions for extended belief or knowledge (e.g., mobile access to Google Search), this does not mean that there is no future for Internet-extended minds or Internet-extended knowers.

From an engineering perspective, the HoloArt case introduces us to some of the challenges confronting the effort to implement systems that support Internet-extended knowledge. In addition to efforts to ensure that online information is suitably poised to influence thought and action (in the manner expected by the folk psychological apparatus of thought ascription), it is also important to ensure that world-involving circuits do not jeopardize the epistemic standing of individuals by fostering the formation of false beliefs. The HoloArt app addresses this concern via a verification technique, called the endorsement step. The inclusion of this step means that it is difficult for human users to be led astray by the informational deliverances of the HoloLens. At the same time, however, the endorsement step need not violate any of the assumptions that underlie the appeal to automatic endorsement in arguments for the extended mind. In this sense, the design of the endorsement step highlights one of the ways in which the “designers and users of new tools and technologies might exercise due epistemic caution while *simultaneously* aiming for the fluid incorporation of those tools and technologies deep into our cognitive repertoires” (Clark, 2015, p. 3374, original emphasis).

One of the virtues of the HoloArt case is that it introduces us to issues and concerns that are not so readily apparent in the original Otto case. In particular, the HoloArt case encourages us to reflect on some of the criteria that have been used to evaluate putative cases of cognitive extension (e.g., the trust+glue criteria). When it comes to issues of availability, for example, the HoloLens device is unlikely to be used on a continual basis. This might be thought to undermine its candidacy for inclusion into an individual's cognitive (or doxastic) system. On the other hand, however, the HoloLens is merely one of a number of devices that could be used to access the *same* body of online information in different situations. Accordingly, there seems to be no good reason to countenance the idea that a *particular* resource (e.g., the HoloLens) needs to be reliably available or typically invoked as part of our evaluation of extended epistemic systems. What matters for extended knowers (or extended believers) is simply the fact that some body of information is available to support the expression of behaviors that are consistent with the folk psychological strategy of explaining/predicting behavior via the ascription of mental states. Thus, while the HoloArt case fails to qualify as a form of extended knowledge according to the traditional interpretation of the availability criterion (i.e., the interpretation afforded by the Otto case), it is much less clear that it fails to qualify as a form of extended knowledge once we direct our attention to a wider ecology of Internet-enabled devices, all of which function so as to service the epistemic interests of the would-be knower. This highlights an important feature of the HoloArt case: it shows how a consideration of technologically-advanced resources might yield something in the way of a philosophical payoff, helping us to better understand what it means for the human mind to escape its cranial confines and leak out into the world.

## Data Availability Statement

Publicly available datasets were analyzed in this study. This data can be found here: https://github.com/ps02v/HoloArt.

## Ethics Statement

Written informed consent was obtained from the individual(s), and minor(s)' legal guardian/next of kin, for the publication of any potentially identifiable images or data included in this article.

## Author Contributions

The author confirms being the sole contributor of this work and has approved it for publication.

## Funding

This work was supported by the UK EPSRC as part of the PETRAS National Centre of Excellence for IoT Systems Cybersecurity under Grant Number EP/S035362/1.

## Conflict of Interest

The author declares that the research was conducted in the absence of any commercial or financial relationships that could be construed as a potential conflict of interest.

## Publisher's Note

All claims expressed in this article are solely those of the authors and do not necessarily represent those of their affiliated organizations, or those of the publisher, the editors and the reviewers. Any product that may be evaluated in this article, or claim that may be made by its manufacturer, is not guaranteed or endorsed by the publisher.

## Footnotes

^1^The source code for the HoloArt app is available via GitHub: https://github.com/ps02v/HoloArt.

^2^See https://cloud.google.com/vision (accessed February 27, 2021).

^3^The specific service that is being used here is the Google Cloud Vision API. While this service can return a variety of types of information about an image (e.g., whether the image contains a human face), the HoloArt app is configured to request information about a specific type of information, namely, Web entities. (See https://cloud.google.com/vision/docs/detecting-web, for more information).

^4^See http://dbpedia.org/sparql (accessed February 27, 2021).

^5^The holographic display panel is rendered in a fixed location within the local environment of the user. Accordingly, it will only appear within the user's field of view if the user is looking at the painting for which the painting recognition process was invoked. This capability relies on the spatial mapping capabilities of the HoloLens device.

^6^A video demonstration of the painting recognition process is available here: https://youtu.be/5Vx9n07aUKE. This video shows the painting recognition process operating in a purely virtual environment (as opposed to a real-world environment); nevertheless, the computational steps associated with the painting recognition process are the same as those used in the context of the HoloArt app (i.e., the app that is deployed to the HoloLens device).

^7^Semantic knowledge is a form of declarative knowledge relating to specific facts (e.g., the knowledge that Harry S. Truman was the 33^rd^ President of the United States) (see Brogaard, 2014).

^8^This analogy with the Otto case serves to remind us that the HoloArt case builds on the original arguments for the extended mind (Clark and Chalmers, 1998). In particular, the claim is that Gustav's beliefs are extended in the HoloArt case, just as Otto's beliefs are extended in the Otto case. Having said this, the differences between the two cases should not be underestimated. The Otto case, for example, is based around a technologically low-grade bio-external resource, namely, a paper-based notebook. The HoloArt case, by contrast, features a complex array of technologically-advanced resources. One of the ingredients of arguments for the extended mind is the so-called parity principle, which was first introduced by Clark and Chalmers (1998). As noted by Wheeler (2011), the parity principle has been subject to a persistent misinterpretation by both friends and foes of the extended mind. The correct reading—according to Clark (2011, p. 451)—is “not that external stuff must work in much the same way as inner stuff if cognition is to depend on extended mechanisms. Rather it [the parity principle] was to probe how we would treat the functional analogs of certain external contributions were they (appropriately) internally relocated.” The application of this principle to the HoloArt case is complicated by the nature of the bio-external resources. In particular, it is difficult to imagine the functional equivalents of the HoloLens and the online services being “internally relocated” or as being subject to in-the-head forms of neural realization. Despite this, however, I suggest that there is no real violation of the parity principle here: if we were to encounter some alien being whose inner workings resembled those of the bio-external features of the HoloArt case, such that the visual perception of real-world objects culminated in the delivery of accurate verbal reports about those objects, then I can see no reason why we would not regard the inner workings as forming part of the *cognitive* architecture associated with such beings.

^9^Note that the HoloArt case is intended to serve as a counterpart to the original Otto case, which is rooted in an appeal to dispositional beliefs. The same is true of the HoloArt case. Accordingly, in talking about Gustav's knowledge as being a form of extended knowledge, I mean this to be taken as a claim about the epistemic status of Gustav's *dispositional* beliefs. These dispositional beliefs are “extended” in precisely the same sense as Otto's dispositional beliefs are extended. In short, we ascribe dispositional beliefs to Gustav and Otto as a means of establishing a predictive and explanatory toehold over the behavior of the two protagonists. Otto thus goes to 53rd Street because he believes (even before accessing the notebook) that this is where MOMA is located; Gustav furnishes us with correct information about a particular painting (e.g., the *Mona Lisa*) because he knows (even before invoking the services of the HoloArt app) something about this painting. In particular, Gustav knows what the *Mona Lisa* looks like, and he is able to link this visual information with bodies of semantic knowledge pertaining to the painting's title and the artist responsible for the painting. At various points in the paper (e.g., the discussion of retrieval latencies in section 4), I will refer to features of the run-time performance of the painting recognition process. This should not be seen as inconsistent with the central focus on dispositional knowledge. The appeal to run-time features is merely intended to evaluate whether it still makes sense to credit Gustav with dispositional knowledge: if Gustav does not possess occurrent knowledge (courtesy of the run-time features of the painting recognition process), then he cannot be said to possess dispositional knowledge either. In short, it is the run-time features of the painting recognition process that underwrite claims about the possession of dispositional knowledge. As noted by Clark (2010a, p. 88), “the very notion of a dispositional belief already makes implicit reference to what would happen in possible run-time situations.” If these run-time situations do not support claims of dispositional belief (and, in our case, knowledge) then it should be clear that we have no basis for claims regarding *extended* dispositional beliefs (or, in our case, knowledge).

^10^Consider, for example, that any hacking-related efforts must be completed within a rather limited temporal window, i.e., approximately 6 s (see section 4).

^11^This is not to say that Gustav is *solely* responsible for determining the truth of his beliefs. The reliability of the larger belief-forming process is one that depends on forces and factors that are external to the endorsement step. Consider, for example, that we could have engineered the HoloArt app to misidentify paintings. That is to say, we could have returned false information about the title and artist while returning the correct image of the painting. In this case, Gustav would implement the endorsement step, acknowledge the match between the actual and virtual paintings, and proceed to endorse the false information delivered by the painting recognition routine. In this sense, Gustav cannot be solely responsible for the truth of his beliefs; we also have to assume that the painting recognition process operates in a reliable manner, i.e., in a way that ensures the performance of the endorsement step does not lead to the formation of false beliefs.

^12^An anonymous reviewer suggested that the internally-situated nature of the endorsement step—the fact that it is being performed by Gustav, or some part of Gustav—undermines the appeal to cognitive extension. The idea, it seems, is that the endorsement step ought to be performed external to Gustav. In response to this, it is important to note that the endorsement step occurs as part of a larger process that includes a mixture of biological and non-biological resources. This hybridity is, I think, perfectly compatible with our contemporary understanding of the nature of extended cognitive processes, and it suggests that even in the case of extended cognitive circuits, we ought not to demand that every part of an extended cognitive routine is located external to an individual. In the original Otto case, for example, there is clearly a lot of work being performed by Otto's biological brain (e.g., visual perception), and this is so even if the larger routine is one that extends beyond Otto's biological borders. The mere presence of some sort of internal goings-on (e.g., visual perception) does not, I think, undermine claims regarding the extended status of the larger circuits that include these internal goings-on. Also note that some of the efforts to combine a virtue epistemological account of extended knowledge with predictive processing accounts of brain function have accorded a significant role to precision weighting schemes (Clark, 2015). These precision weighting schemes are all realized by internally-situated neural mechanisms, but such forms of intracranial localization are not seen to be inimical to extended knowledge (see Clark, 2015, p. 3771).

^13^Recent attempts to develop a virtue epistemological account of extended knowledge have raised the possibility of a dilemma that centers on the extent to which “an agent consciously encounters some new resource as an ongoing object for various forms of epistemically hygienic practice…” (Carter et al., 2018b, p. 334). The source of this worry can be traced to a contrast with biological memory. In particular, Clark (2015, p. 3763) notes that “biological memory is not typically subject to agentive scrutiny as a process at all, much less as one that may or may not be reasonably judged to be reliable by the agent.” One problem, here, is that, in earlier work, Clark appeals to the commonsense functional role of a bio-external resource in underwriting claims of mental extension. As noted by Andrada (2021), it is hard to see why this commonsense functional role would be impugned by a shift in the distribution of attentional resources or a change in the phenomenological experiences of a subject. In respect of this issue, she writes: “…it is easy to see that playing this [commonsense functional] role should be entirely compatible with an active pursuit of epistemic hygiene, i.e., with focal attention and cognitive awareness. For it is unclear why such traits and attitudes should in any way hinder the storing and retrieving of (action-guiding) information” (Andrada, 2021, p. 4698).

^14^It should be noted that the Crossed-Out Otto case does not echo all the features of the HoloArt case. For the Crossed-Out Otto case, it is natural to assume that Otto implements the crossing-out activity prior to the point at which information is accessed. In the HoloArt case, however, there is no earlier “crossing-out” activity; rather, the “crossing-out” occurs at the point at which information is delivered by the HoloLens. At this point, Gustav implements the endorsement step and either ignores or accepts information based on the match between the holographic painting and the real-world painting.

^15^Note that the appeal to latency-related issues serves as an effective antidote to worries about the notorious problem of cognitive bloat (e.g., Allen-Hermanson, 2013). If Gustav fails to qualify as an extended knower due to the fact that it takes (e.g.,) >10 s to retrieve painting-related information using the painting recognition process, then, in all likelihood, a longer latency routine (e.g., a trip to the library, the use of an art textbook, or a phone-a-friend strategy) will not be sufficient for extended knowledge either. In this sense, there is no need to worry about counter-intuitive extensions of Gustav's mind. We can accept that Gustav knows something about art, courtesy of the operation of a reliable, low-latency, world-involving circuit, without falling into the trap of thinking that *every* form of world-involving circuit will be sufficient to ground claims of extended knowledge.

^16^This does not mean that a consideration of resources is irrelevant, of course. The resources that deliver information to an individual must lie external to the individual; otherwise, claims about *extended* belief are apt to be called into question. Furthermore, claims about *Internet*-extended belief (and thus knowledge) rely on the idea that at least some of the bio-external resources will qualify as *online* resources.

^17^See, for example, the Smartify app (https://smartify.org/), which is available for Apple and Android devices.

^18^An anonymous reviewer noted that much of the information relevant to Gustav's beliefs is provided by the DBpedia service and this service remains a common element of the painting recognition process regardless of the specific nature of the “frontend” device (e.g., HoloLens or smartphone). Perhaps, then, the proper target of the availability criterion ought to be the DBpedia service, since this service (and the associated database) appear to be playing a role similar to that afforded by the notebook in the original Otto case. There are couple of things to note here. The first is that the DBpedia service is not the only source of belief-relevant information in the HoloArt case. Relative to the way the painting recognition process works, the machine vision service plays a crucial role in identifying the painting's title. The DBpedia service is thus not the *primary* source of all the information that is relevant to Gustav's painting-related beliefs. Rather, there is a distribution of responsibility between the online constituents of the painting recognition circuit: The machine vision service “retrieves” information about the painting title, and this information is then used to cue the “recall” or retrieval of other information via the execution of a query that is posted to the DBpedia service. A second point to note is that claims about the substitutability of the “frontend” devices (e.g., the HoloLens) are just as applicable to the “backend” resources that are located in the online environment. Consider, for example, that both the machine vision service and the DBpedia service could be substituted with alternative services, providing these substitutions did not affect the functional profile of the painting recognition process. There is, in this sense, nothing special about the machine vision service and DBpedia service. Different token instantiations of the painting recognition process may rely on the same or different services, but providing all these services succeed in maintaining the functional poise of bio-external information (in a manner that supports the ascription of belief and knowledge), then these variations will not materially alter Gustav's thoughts and actions. What matters, I suggest, is not the reliable availability of specific resources; it is the reliable presence of the *information* that sustains epistemically-relevant performances.
